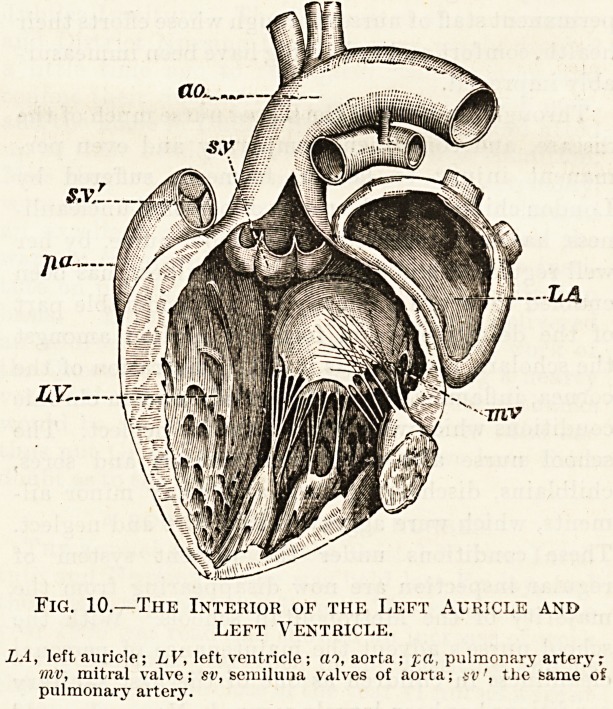# The Hospital. Nursing Section

**Published:** 1906-03-10

**Authors:** 


### The Hospital.

### Nursing Section.

Contributions for "THE HOSPITAL," should be addressed to the EDITOR, "THE HOSPITAL" NURSING SECTION, 28 & 29 Southampton Street, Strand, London, W.C.

No. 1,015.—VOL. XXXIX. SATURDAY, MARCH 10, 1906.

### Notes on News from the Nursing World.

### THE SISTERS' QUARTERS AT MILLBANK HOSPITAL.

IT having been stated that improvements of an important character are to be made in the sisters' quarters at the Military Hospital, Millbank, we are in a position to say that, as a matter of fact, comparatively little has been done during the last few months. That little, however, is in the right direction. According to the original arrangement the Home was to have been roofed by last Christmas, but delay has arisen owing to extra rooms having been added at the top. It will be remembered that this was Queen Alexandra's own suggestion for providing the sisters with better accommodation than was at first proposed. It is premature at present to indicate definitely the exact purpose for which these rooms are intended, because the building operations are still far from advanced, and no one is allowed inside the walls. There is yet, however, ample time for further developments as, at the very earliest, the staff will not move in to the Home before the end of the year, and it will probably be later. Meanwhile, the sisters continue to go backwards and forwards to the hospital from their temporary quarters in a pleasant square nearly half a mile distant from the scene of their duties.

### IRISH NURSES AND THEIR GRIEVANCES.

THE hardships endured by Irish nurses are being set forth in the Irish daily press just now, but we regret to say that, having perused a number of the letters which have been published, chiefly from anonymous writers, we are not able to form a clear idea as to the nature of the alterations desired. Some of the correspondents deal largely in vague generalities, and they are profuse in declarations of sympathy for the alleged victims of over-work, but they do not make any practical proposals; while the nurses who contribute to the controversy fail to cite any specific grievance with which it would be possible to deal. If any of our readers on the other side of St. George's Channel will send us authenticated details which show that in a particular instance, at a particular institution, nurses are over-worked or ill-treated, their communications will be regarded as strictly confidential, and we shall be pleased to do our best, by publishing the facts and our own opinion of them, to bring about reform. The mere piling up of the agony in the lay press is much more likely to do harm than good.

### AN ATTACK ON THE CENTRAL MIDWIVES BOARD

AT a general meeting of the members of the National Association of Workhouse Masters and Matrons, held in Manchester the other day, a report was presented on the regulations of the Central Midwives Board. It having been stated that, as they now stand, it is impossible to get a sufficient number of cases in many Poor-law infirmaries to qualify for the necessary certificate, Dr. Rhodes affirmed that the "Central Midwives Board do not, in his opinion, understand Poor-law institutions in the slightest degree." He went on to say that "not only small, but large Poor-law institutions have been treated with contempt by the Board, and that unless the regulations are altered, the whole question will have to be raised in Parliament." It is obviously open to anyone to raise the whole question in Parliament, but we protest against the statement of Dr. Rhodes that the Central Midwives Board treat Poor-law institutions "with contempt." The Board, like other public bodies, is not infallible, and in its brief career it has no doubt made mistakes. But the charge brought against it by Dr. Rhodes cannot, we believe, be sustained. At any rate, as he preferred it, he should have attempted some kind of justification instead of apparently assuming that he had settled the question by a sweeping assertion.

### THE INTERFERENCE OF THE WORKHOUSE MASTER.

AT the last meeting of the Exeter Guardians a letter was read from the superintendent nurse at the infirmary complaining of the interference of the master of the workhouse. Acting, as he stated, on instructions, he insisted upon checking the heat of the sick wards from time to time, a course of procedure which the superintendent nurse affirmed was unpleasant and tended to lessen her authority with the patients. She added that she was responsible to the medical officer for the heat of the wards, but not to the master of the workhouse. In the discussion on the letter several Guardians endorsed the views of the superintendent nurse, and others said that the authority of the master must be upheld, the matter being finally referred to the Management Committee. In this case, however, it should at once have been decided that the master has no *locus standi*. The new infirmary at Exeter is distinct from the workhouse, and the superintendent nurse is responsible, not to the master, but to the medical officer, who will probably take care that in future no interference is permitted in the domains over which he ought to have supreme authority. The question of heating the wards is one that should certainly be left to the superintendent nurse, and it is for her, subject in this as in other respects to the approval of the medical officer, to determine the quantity of fuel to be consumed.

### ENGLISH NURSES IN ROME.

The seventh annual meeting of the General Committee of the Anglo-American Nursing Home in Rome was held on the 23rd of last month at the British Consulate. Commenting on the report submitted to the meeting, the Chairman, Mr. A. Chenevix Trench, pointed out the progress of the institution since its establishment. He also drew attention to the outlay about to be incurred for the enlargement of the Home and for the isolation pavilion to be built on the recently purchased land, at a cost of £2,400, exclusive of heating, lighting, and furnishing. Looking over the report, which has been sent to us for criticism, we notice that 18 nurses were engaged for the season last year, of whom two were on the staff the previous year. It is stated that the requests for nurses in January, February, and March were considerably in excess of the number available, and that many applications for their services could not be complied with. We should think that it would be to the advantage of the institution if the Committee did not seem to be compelled to practically change the personnel of their staff each season. Mention is made in the report of a desire on the part of Professor Durante to secure the services of two English nurses to superintend his pavilion at the Policlinico with Italian nurses under them, but it appears that the scheme has been for the present postponed. The committee congratulate the directress "on her efficient management of the Home, and on the success of her constant efforts to render it useful."

### THE MORAL OF A SAD CASE.

It cannot be said that the unfortunate staff nurse at the Holborn Union Infirmary who a few days ago committed suicide by taking carbolic acid was too young to bear the strain of the examination which she seems to have feared. This poor woman was 36, and it might reasonably have been concluded that the by no means formidable ordeal of an examination would have been without terrors for her, especially as the medical superintendent of the Holborn Infirmary gave evidence at the inquest to the effect that she was one of his most promising pupils, and could have passed easily. As the matron told the coroner that she knew of no trouble on the mind of the deceased, it can only be concluded that she was one of the number—we are afraid the inevitable number—of persons who take up nursing without realising that both physical and mental strength, are essential conditions, not merely of success, but also of contentment. No one possessing these two essential qualifications could have written, as she wrote, that "there seems nothing to live for here" when the wards are full of suffering humanity.

### THE GIFT OF A HOME TO WALSALL.

It will be remembered that a floral bazaar held at Walsall in October yielded a huge sum of money. At the annual meeting of the Walsall Victoria Nursing Institution, mention was made of this remarkable success in the report, and the chairman, who moved its adoption, referred with gratitude to the organisers. The exact sum handed over to the Treasurer was £2,046 8s. 11d. But the institution is in luck's way, for as soon as the chairman had congratulated the meeting on the support accorded to it, Mr. Graham Leckie, who spoke next, announced the presentation of a new home. The generous donor is Mr. John Leckie, of Crosby Lodge, Torquay, who has chosen this mode of perpetuating the memory of his wife in Wallsall, where she formerly lived. The monetary value of the gift is between £2,000 and £3,000, and includes an endowment fund for repairs and enlargement should the house acquired be found too small for the nursing staff, which consists of the lady superintendent, three district, and eleven private nurses. The only fly in the ointment is that there was a small loss on the private branch of the institution last year, and it seems a pity that the Committee, in view of the splendid help given them, do not decide to run the district work by itself.

### TWO CLASSES OF PROBATIONERS.

The result of the criticism on Dr. Caleb Powell's nursing scheme, which he has invited the Guardians of North Dublin Union to sanction, is that he proposes to modify it to the extent of having two classes of probationers. Dr. Powell suggests that there should be one class to serve for a term of two years before they receive a certificate of training; and another to serve for a term of three years. He confesses that he has been induced to adopt the alternative course of three years because he knows that there are some public bodies who will not employ a nurse unless she has a certificate of at least three years. He proposes that the two-year probationer shall pay a fee of 20 guineas and the three-year probationer one of 12 guineas, the former also supplying her own uniform, and the latter being paid at the rate of £8 per annum for her second and third years' service. But surely the simpler plan would be to adopt the terms of three years for all the nurses trained under the auspices of the North Dublin Guardians.

### A LONDON NURSING ASSOCIATION IN DIFFICULTIES.

At the annual meeting of the North London Nursing Association last week, complaints were made of the apathy of the public with respect to its needs, and it was stated that the debit balance at the end of the year was nearly £230. This does not speak well for the Islington people in whose interests during twelve months the ten nurses of the Association paid 35,000 visits; nor particularly well for the manner in which the Association is managed. A little more enterprise might be shown, for example, in sending out invitations to attend the annual meeting, of which we received no notice. This is an omission that points to lack of attention to the interests of the organisation. At least we imagine that the subscribers to the North London Nursing Association desire all the publicity they can obtain for their work; and in spite of official remissness, we gladly urge the claims which it has upon the charitable. At the meeting the lack of support of the clergy and ministers of Islington was specially deplored, but it is very probable, as the Rev. J. M. Hanks said, that the reason of this is that they do not know what is being done in their midst. Persistent efforts should be made at once to deprive them of the power of advancing the plea of ignorance.

### THE THREE MONTHS' TRIAL.

The Aston Guardians have decided that in future candidates for the position of probationers shall serve for three months, instead of one month, on trial before appointment. We entirely agree with the view that one month's trial is too short a period in which to judge of the fitness of a girl for the vocation of a nurse. It is equally true that it is difficult, if not impossible, for a girl to judge, in one month, how far she is qualified for the discharge of the duties a nurse is required to perform. The trial of three months should be the rule at all hospitals and Poor-law infirmaries which aspire to be regarded as approved training schools.

### PAYMENT IN KIND.

At the annual meeting of the Leeds and District Nursing Association attention was drawn to the fact that last year the sum of £48 was expended on tramcar and railway fares, the secretary observing in her report that this was sufficient to support a nurse for a year. Subsequently the Chairman, an alderman of the city, said that he would bring the matter before the Tramway Committee of the Leeds Corporation, with a view to obtaining some recognition of the services of the nurses. It cannot be contended that associations have a right to expect that their nurses should travel either by tram or rail free of charge; and while we do not, of course, deprecate concessions in this direction, it would be better if the respective companies would show their appreciation of the work done by the nurses by a regular subscription. Other companies unable to acknowledge the services of the nurses by payment in kind might then be moved to emulate their example.

### BAD ACCOMMODATION IN BIRMINGHAM.

The most striking feature in the report of the Birmingham District Society, which was adopted at the annual meeting last week, presided over by the Lord Mayor, is that the building in Newhall Street, where the nurses reside, is in very bad structural repair, and the accommodation quite inadequate to the needs of a modern district nursing home. Although, as we regret to learn, the financial position is not flourishing, there being a balance of £237 on the wrong side last year, the need of the nurses belonging to the Society being properly housed is so imperative that steps should be taken without delay. It is unreasonable to expect that the nurses will continue to discharge their duties satisfactorily so long as they are denied ordinary home comforts; and we are glad that the General Committee of the Society have been authorised to arrange with the trustees to provide and equip a suitable building.

### DEATH OF A CRIMEAN NURSE AT SMYRNA.

The death occurred at the British Seamen's Hospital, Smyrna, on February 24, of Mrs. Eliza Turnbull, who at the time of the Crimean war was one of Miss Florence Nightingale's nurses in the hospitals at Scutari, and helped to tend the wounded soldiers. The old lady, who was 88 years of age, was a frequent inmate of the Smyrna Hospital during the last years of her life, and she was much beloved by the sisters and nurses. She was very sweet and gentle, and often advised the young members of the staff to be more tender to the sick. She liked to describe how excellently the nurses at the Crimea did their bandaging, and she could remember many incidents of the war and its horrors. Her maiden name was Limmes, and she was the daughter of a merchant captain. She is buried in the English cemetery at Smyrna, not far from the memorial erected to the heroes of the Crimea who died of their wounds in the hospital. "Granny" Turnbull, as she was generally called, remembered Miss Nightingale, and frequently used to tell the nurses of "the good and gentle lady who was so beloved by all her soldier patients."

### OPENING OF A HOME AT GRIMSBY.

On Thursday last week the new Home for Queen's Nurses at Grimsby was opened by Miss Amy Hughes, general superintendent of the Queen's Jubilee Institute. The Committee of the Grimsby and District Nursing Institute very wisely decided a little time ago to relinquish paid nursing and confine their duties to the nursing of the sick poor, and the home in Dudley Street was taken and fitted up as their headquarters under the new condition. Miss Hughes, in performing the opening ceremony, wore her superintendent's uniform, and delivered an excellent address, in the course of which she warmly congratulated the committee of the institute on placing the work of the district on its own basis. Later in the day Miss Hughes delivered an address at King's Hall on the general work of the Queen's Nurses, and in acknowledging a hearty vote of thanks, observed that the best vote of thanks would be the securing of another nurse. She has thus put Grimsby on its mettle, and we have little doubt as to the result.

### A BONUS FOR BRISTOL NURSES.

The annual meeting of the Bristol Nurses' Institute and Nursing Home was held last Friday, and there was a good attendance. The report for the year 1905 was read and showed an increase of work on the previous year both in the Institute and in the Home. At the close of the year 41 nurses were at work. The private nurses did 1,591 weeks' work, and 252 weeks' work was done in the Home. It was stated that the small fee of two guineas a week charged to patients coming into the Home and whose means did not allow of their paying more, had been much appreciated in many cases. The balance-sheet showed that enough profit had been made to allow a bonus to be given to each nurse of over one year's standing, varying in sums from £3, to £12 for nurses who for twenty years had worked for the Nurses' Institute; this was in addition to the 10 per cent. paid on each nurse's earnings.

### The Nursing Outlook.

"From magnanimity, all fears above;
From nobler recompense, above applause,
Which owes to man's short outlook all its charm."

### THE QUEEN'S NURSES AND SCHOOL CHILDREN.

One of the most inspiriting and helpful of books might be written on the far-reaching effects for good to the whole nation which has resulted from the voluntary efforts of intelligent citizens of both sexes. Miss Susan Lawrence, when elected to the School Board for London, devoted much of her energies to looking after the physical well-being of the children in the schools. She was largely instrumental in establishing the London School Nurses' Society, without which effort the school nurse, in London, at any rate, would probably not have been tried. But for this society, the school nurse would certainly not have been established upon the satisfactory basis upon which she now rests under the Education Committee of the London County Council of which Sir William Collins is the able chairman. To-day children who attend the public schools throughout London have the benefit of a permanent staff of nurses through whose efforts their health, comfort and well-being have been immeasurably improved.

Through the work of the school nurse much of the disease, and consequent temporary and even permanent injury to health, formerly suffered by London children, as the result of personal uncleanliness, has been removed. The school nurse, by her well regulated and constant watchfulness, has been enabled to secure a remedy for a considerable part of the defective vision formerly noticed amongst the scholars, resulting as it did in ulceration of the cornea, inflammation of the eyelids, and in chronic conditions which were aggravated by neglect. The school nurse also deals with wounds and sores, chilblains, discharging ears, and many minor ailments, which were aggravated by dirt and neglect. These conditions under the present system of regular inspection are now disappearing from the majority of the Metropolitan schools. With the school nurse's advent the maintenance of personal cleanliness in children as one of the first sanitary conditions has been largely secured. No words could exaggerate the importance of the results which have been collectively secured by the appointment of the school nurse.

It was of the first importance that the nursing staff of the Queen Victoria Jubilee Institute for Nurses should be enlarged and extended, until it was strong enough to secure the appointment of a district nurse for every town and country district throughout the country. We are glad to see that the Council of this Institute, on the motion of Mr. Archibald Williamson, M.P., have made a strong representation to the Board of Education, pointing out the advantages to the children and to the community which are derived from the attendance of hospital-trained nurses on the children in elementary schools. The Council ask that the Board of Education shall provide in the new Education Bill that any impediments to grants being made by local educational authorities to district nursing associations may be removed, where, on grounds of efficiency and economy, it is considered advantageous to have this work done by district nurses, who are already in constant touch with the poor in their own homes, and whose general nursing experience is continually kept up to date. We cordially support the recommendation of the Council of the Queen Victoria Jubilee Nurses' Institute, because it is essential for the welfare of the children in the public schools, and therefore for the whole nation, that every school shall have the services of a school nurse on some such system as that of the Education Committee of the London County Council, which in practice has proved so excellent in its results.

The action just taken by the Council of the Jubilee Institute follows the lines which we took up in regard to the London School Nurses' Society, which has resulted in the adoption of the school nurse system by the education authority throughout London. Before the change was made, in London, it was found that ninety per cent. of the children from the public schools had vermin-eaten heads, and many others had their underclothing stitched on, so that the little bodies got no chance of being washed. Probably a similar state of affairs exists still, in many places where the local education authority has not yet organised a system of school nurses. This makes the adoption in its new Education Bill by the Government of the scheme proposed by Mr. Williamson the more urgent, for it is evident that until the children have every facility for possessing healthy, well developed and cleanly bodies, all attempts to train and develop their brains must be largely ineffective. No thoughtful Minister can hesitate to support the recommendation of Mr. Williamson, seeing that the provision of the necessary baths and appliances, together with a staff of trained nurses, as in American and Continental cities, will not amount to an extra annual charge of more than a few thousand pounds, whilst the capital outlay involved will be so little as to be relatively inappreciable. We cannot believe that Mr. Birrell or the Government will ignore the claims of the children's bodies in public schools, for if they do, they must cripple the educational progress of the children, and minimise the powers of the teachers too.

### The Care and Nursing of the Insane.

By Percy J. Baily, M.B., C.M.Edin., Medical Superintendent of Hanwell Asylum.

### I.—ANATOMY AND PHYSIOLOGY.

(*Continued from page* 317.)

### The Heart and Blood-vessels.

In no part of our bodies does the blood come into actual contact with the tissue elements or cells,* but it is contained entirely within a system of closed tubes which are collectively spoken of as the blood-vessels. These blood-vessels are found in every tissue in the body (except cartilage) and through them the blood is forced by the pumping action of the heart. The simplest form of heart—that of the cockroach for example—may be aptly compared to a Higginson's syringe. It consists of a simple tubular dilatation with muscular walls, so that when these walls contract the blood which is contained within it is forced out, just as the contents of the bulb of a Higginson's syringe are forced out by the pressure of the hand. Each end of the bulb of the syringe is provided with a valve and these valves allow the water to move only in one direction through the syringe.

The human heart (fig. 9) is necessarily a more complicated structure than this simple one of the

Fig. 9.—The Human Heart.

*RA*, right auricle; *RV*, right vent icle; *LA*, left auricle; *LV*, left ventricle; *AO*, aorta; *PA*, pulmonary artery; *VC*, vena cava superior; *PV*, pulmonary veins.

cockroach, but it is arranged on quite the same principles—that is to say it is like the Higginson's syringe, a force pump, but instead of having only one chamber it has, as we shall presently see, four.

It is a hollow conical or pear-shaped muscular organ and lies within the thorax behind the breast-bone, resting upon the upper surface of the diaphragm. The greater portion of the heart is behind the breast-bone, but the point or apex of the cone projects beyond the left edge of the sternum and can be felt beating between the fifth and sixth ribs below the left breast. The thick end of the cone—the base of the heart as it is called—is the highest part as it lies in the chest and it is at this part that the great blood-vessels enter and leave the heart. It must not, however, be supposed that the heart rests upon its apex, it really lies upon its right side so that the apex points downwards and to the left, while the base is directed upwards and to the right, nor does it entirely depend upon the diaphragm for support since the great vessels which enter or leave it at the base, help to suspend it. In size it is generally said to resemble that of the closed fist of the person to whom it belongs and its weight is about nine ounces.

It is enclosed within a loose fibrous bag which is called the pericardium. This is attached around the base of the heart at the origin of the great blood-vessels, so that the apex of the heart lies quite free within it. The inner surface of the pericardium and the surface of the heart are smooth and glistening, and are separated from each other only by a very thin layer of fluid—the pericardial fluid. The object of this arrangement is that the heart, which, of course, moves every time it contracts, may not be impeded in its action by rubbing against the surrounding organs. Throughout the body a similar arrangement is found wherever one part moves over another.

The interior of the heart is divided by a complete partition, which runs from the base towards the

Fig. 10.—The Interior of the Left Auricle and Left Ventricle.

*LA*, left auricle; *LV*, left ventricle; *ao*, aorta; *pa*, pulmonary artery; *mv*, mitral valve; *sv*, semiluna valves of aorta; *sv'*, the same of pulmonary artery.

apex, into a right and left chamber. These two chambers have no communication with one another, so that the blood which is contained within one chamber is entirely separated from that which is contained within the other chamber.

Each of these two chambers is again divided into an upper and a lower part by a partition, which is, however, not complete, as there is an opening through which the blood passes from the upper chamber into the lower one on each side. Thus we see that the interior of the heart is divided up into

* This statement is not strictly correct, since in the spleen and in the walls of the blood-vessels this does occur.

four chambers, an upper and a lower one on each side, and that while there is no direct communication between the right and the left chambers, there is such communication between the upper and lower chamber on each side.

The interior of these chambers is lined by a membrane (the endocardium), which forms curtain-like folds around the openings between the upper and the lower one on each side (fig. 10). Of these curtains or folds (cusps) there are two on the left side and three on the right, and they hang down into the lower chamber. They are loosely tied down to the muscular walls of the lower chamber by fine but tough thread-like cords which are attached to their lower margins.

The upper chambers on each side are called the auricles, right and left, while the lower ones are the ventricles, also right and left; and the membranous curtains which hang down around the openings leading from the auricles into the ventricles are called valves. That on the left side with only two curtains is called the mitral valve, while the one on the right side with three curtains is called the tricuspid valve.

(*To be continued.*)

### The Nurses' Clinic.

### REST CURES: THE MENTAL POINT OF VIEW.

At the present day, when cases of nervous breakdown and overstrain are terribly prevalent, the so-called Weir-Mitchell treatment, or a rest-cure in some form, is frequently prescribed. For the carrying out of this treatment, however, the usual hospital training does little to prepare a nurse, and it may be that we should see fewer failures as a result of these cures, if nurses had studied and thought over this branch of nursing more thoroughly before undertaking it. I have heard it said that "anyone can nurse a rest-cure case," but a little consideration will, I think, show how wrong is this idea.

### Condition of Rest-cure Patients.

Now, many of these cases, when they do consent to be treated, have either lost for a time the power to help themselves, or feel that they are on the verge of losing it. They are unbalanced; mind, soul, and body no longer work together harmoniously under the direction of the individual will, but rebelliously war with one another or refuse to work at all. In this condition do these weary persons come to us for treatment, and it is our business to help them to face life again, to stand once more on firm ground, and drink in for themselves sufficient strength and inspiration to enable them to live their lives sanely, strongly, and usefully. Now I think that too often we turn all our attention to helping only the physical condition. True, this is of great importance, though the easiest part of the work, and occasionally when a person's physical condition has improved, the mind seems to regain balance of itself, so marvellously do the different parts of our being react on one another. But this is not, I think, usually the case, and one sees too many patients who, at the conclusion of their treatment, were considered successes, relapsing shortly into a pitiable moral condition of apathy and selfishness. In considering the question of the nursing treatment of these cases, I shall discuss it firstly from the more entirely mental point of view; secondly from a physical point of view, although these two aspects must necessarily touch each other at all points.

### The Personality of the Nurse.

The nurse who undertakes the care of nervous patients will often have to bear severe mental strain. She ought, therefore, to be well balanced herself, and should have studied life sufficiently to have in her heart "a reason for the hope that is within" her. For she will have to live with and battle with morbid, distorted views of every kind, and her very presence should breathe forth a knowledge of, and belief in, the existence of a law of love and order. Above all, she needs to possess great insight into character, and wide-minded sympathy. That these two qualities are essential will be clear if we consider a few of the types of people that the nurse will have to do with.

### Types of Patients.

Her patient may be a highly sensitive person, one who has lived a life of culture and wide interest, in whom the virtues of courtesy and self-control have hitherto seemed part of the nature; yet now, when the nurse—a stranger—first makes her acquaintance, her horizon has become so narrow that it seems bounded by the petty aches and pains and wants of her own body, and her mind is unable to think or feel beyond that limit. In order to help such a one, the nurse must be able to discern the true nature beneath the present self-absorbed mask, and must make her patient feel that she recognises the real self and is willing and able to help that self to reassert itself; for often the keenest torture and greatest depression are caused to such natures by the feeling that they are unable to show their real selves, or to rise to what they have hitherto been, and they need the wisest sympathy. Or again, she may have a patient who has hitherto been keenly alive to the beauty and joy of life, to whom religion has been a living source of inspiration; but now, she can see no light, feel no love; to her the universe seems deathly cold and dark, and she feels herself to blame for all her trouble. Through this veil, once more, must the nurse's sympathetic insight pierce, to rightly discern the type of soul behind it. Often such cases need no rousing, the condition being almost a natural sequence to some intense strain. Time and patience, rest and light and warmth are great healers for such worn souls, and often their intense depression taxes a nurse's patience to the utmost. Yet only love can heal them; love clear sighted enough to see and seize that right moment when, after sufficient rest, the dulled soul must be helped to clearly see that it stands where two ways meet, and can, if it will, now choose and be whole, thus through a mighty effort saving itself from utter breakdown. It is the nurse's part wisely to prepare her patient for this moment, both by resting, toning, and strengthening the whole physical organism, and by surrounding her with an atmosphere of will, hope, and courage.

### The most Trying Patient.

Let us briefly consider one other type of patient, a type which may oppress and try some nurses more than any other. I refer to those indolent, flabby, selfish, and often vulgar-minded women, who seem to have nothing definite the matter with them, yet declare themselves unfit for any exertion. Such patients are often, though not always, of very shallow mental capacity, and are usually quite content to be invalids and to live a parasitic life, feeding on the strength and kindness of any who will minister to their

**THE NURSES' CLINIC**—*Continued.*

bodily wants. Trying indeed is it to nurse women of this type, and, in weary moments, one may be tempted to ask oneself whether it be not waste of life and strength to freely pour them forth for one who does not even wish to get better. But here, once more, do those great needful qualities of sympathy and insight guide us to a truer view. For however apparently physically sound, the very lack of nerve force, of will, and of refinement, show that there is something wrong in the working of that complex whole which we call a human being,—some essential part is not doing its work, and consequently the harmony is disturbed. It is our part, as nurses, to seek to learn, by experience, thought, and sympathy, how such a condition may best be remedied, how to do our little share of fanning into flame that spark of God which seems buried and asleep in some of these patients. For most of us, our eyes are not yet clear enough nor our hearts full enough of love to understand the true reason for these conditions. We have to work greatly in the dark, very humbly, learning from each patient whom we are called upon to serve, remembering that as surely as one of us is entrusted with the temporary cure of some sick soul, so surely is there something that that one, and no other, can do for her.

### The Patient's Mental Atmosphere.

Most of these cases are intensely sensitive to the mental atmosphere surrounding them. They may possibly preserve a uniformly apathetic expression for days and weeks, but by very careful study we shall discover that they always note "what mood the nurse is in," and that they observe details of every kind, though often quite silent about them at the time. These details have an enormous effect upon them, for their judgment is unbalanced, and their nerves usually strained and hypersensitive. The room in which the case is to be nursed should have a very special cleaning and airing before occupation and should, if possible, be flooded with light and sunshine. Changing the arrangement of the furniture will often alter the "feel" of a room, to which I have frequently found these patients very sensitive. As the nurse will have to pay very great attention to her own attitude of mind and must keep herself balanced and "in tune," and her thoughts free from selfishness and morbidness, it is imperative that she should have certain definite hours out of the sick-room, such hours being spent in whatever way brings her most true refreshment. This can be done if the massage treatment be carried out by a masseuse who is a trained nurse and who sufficiently understands the case to take responsibility during the nurse's absence. The nurse will find that ten minutes a day spent alone in quiet thought for and consideration of her patient's true condition and requirement, will aid her very greatly in her work.

### Patient's Susceptibility to Colours.

Certain colours have a markedly soothing, stimulating, or irritating effect on certain people, and by study we can discover what colours will be likely to prove useful in individual cases. They can be introduced by means of washing covers, lamp shades, and above all by flowers. Flowers and growing plants often prove the greatest aid to the nurse in her work. As her patient gradually begins to regain the power of taking interest in affairs outside herself, the nurse must be prepared to tactfully suggest suitable topics, for at first the patient will only be able to listen for a few minutes to something which she thinks interests the nurse, she will have no power to suggest or consider any subject for herself. The wider the interests and sympathies of the nurse, the easier will be this part of her work, for her patients will vary very greatly in their tastes and pursuits. In the case of those who possess a fairly good intellectual capacity, yet whose nerves are thoroughly tired and irritable, and who are impatient of the tedium of a rest-cure, it is well at first to help them to be content to take an interest in small humdrum matters of daily routine, and thus give their brains a rest. They will generally let themselves do this if they are assured that when the treatment is over the power to think and to follow their usual interests will be stronger than before. These patients need wise nursing, for, on the one hand, our aim must be by over-feeding and the establishment of a rhythmical sick-room routine to induce a temporary condition of drowsiness, and on the other hand to prevent them losing heart, and fearing that they will remain permanently lethargic.

### A Preliminary Step to the Cure.

I have found that half-an-hour's conversation with these patients at the very beginning of the treatment is a wise plan. Let the patient glean an idea of what kind of woman the nurse is and what her ideas as to the aim of a rest-cure are. If once she trusts the nurse half the battle is won. If she undertake the cure because she feels she must, yet loathing it in her heart and secretly rebelling, the chance of a successful issue is small, except in the case of a young girl who has no experience to help her and to whose reason one can often better appeal when the physical condition has improved. Persuade the patient at the beginning to voluntarily put her own will and judgment in abeyance for a season, and show her how this attitude, when taken voluntarily for an unselfish reason, may be a strong and not a weak one. Then, having won her co-operation, let the nurse take her own stand firmly, and without being tyrannical, act and judge for the patient until she be more rested, helping her then to gradually reassume her own work of self-rule. A promise given in good faith to rouse and wake up a person should she remain indolent and lazy as a result of the cure, will often quiet her scruples and help her to give herself up willingly to the treatment. I have dwelt on this point at some length, for I believe that the mental attitude of the patient to the treatment and to the nurse has an enormous effect on the result of the cure, except in those acute cases in which the patient is past thinking or caring what happens to her.

### Incidents in a Midwife's Life.

### MY FIRST TWINS

It was a cold, wet day in November, and I was training to be a midwife in one of the large maternity hospitals. I was just beginning to have a little more confidence in myself, for the week before I had been sent to my first patient alone, since when I had delivered two or three normal cases.

Never shall I forget that first case; only those who have experienced it can have any idea of the feeling. To know that you are going to a patient who perhaps is very far from being normal (even the normal cases frighten one at first), to know you are probably a long distance from a doctor, and that the patient has entire confidence in you. It is agonising to any nurse who is at all of a nervous temperament.

However, on the day I am alluding to a woman came to the hospital in a very excited state of mind, wanting a nurse "at once." Her neighbour, who lived in "—— Street, four stairs up, second lobby, third door to the right, was awful bad."

On inquiring how long she had been ill the answer was, "Oh, nurse, she's been terrible bad all night and all day yesterday; come quick as ever you can," till I began to think the baby was sure to be born before I could arrive.

In about two minutes I was tramping along in the pouring rain with my escort, hearing all her own family history and all the latest murders on the way.

We arrived at —— Street about 10.30 A.M., and I toiled up the four flights of filthy stairs, to find my patient walking about the floor as best she could in the small space. Her face and hands looked as though they had not seen soap and water for twelve months, and her surroundings were almost indescribable; dirt, and poverty, and misery reigned everywhere.

The room was so small one could hardly turn round. The bed stood immediately behind the door, and, with the exception of a very small space just sufficient to accommodate a chair, the rest of the room was taken up with large cages of doves, who made a terrible noise, and nearly drove me frantic all the time I was there.

I began to make preparations, and found the patient was going to lie on the bare mattress, with very little to cover her. The only help I could offer under these circumstances was to spread my mackintosh sheet over the bed, on the top of which I placed several newspapers, borrowed from the neighbours.

The bed was so high that I had to stand on a wooden box to reach, though I am by no means short—in fact, considered rather tall. It was also quite dark in the recess where the bed stood, but this was perhaps as well, as I might have seen more than I cared to. On making the necessary examinations I found that it would not be very long before the baby was born; so after getting everything ready, I settled down to wait. In the meantime the patient and her friend regaled me with the history of all their former babies born into the world.

I discovered, among other interesting news, that my patient had not had a baby born alive for ten years, and always had very bad times; in fact, "the professor's always had to come to her in the end," a statement which caused me to quake inwardly, and to wonder if my diagnosis was all right. However, I reassured the patient, and told her I thought she would be rewarded this time with a living child, for "I had never brought a dead baby into the world, and didn't intend to."

The one pleasing feature of that day's work was that woman's look of relief, and the entire confidence that she placed in me. Little did she know what I was suffering, though I looked so calm and composed.

Dinner time came, and it was wearing on to tea time; still there seemed to be little or no progress.

I was beginning to feel a little hungry, and to wish that I was safely back in the hospital, with everything over.

I was pressed by the kindly neighbours to have tea, and, much as I longed for some, I could not possibly fancy it.

I was nearly sickened with the dirt all round, and the women sat gossiping all day, never attempting even to clean up a little. One very stout woman, who also had not handled soap very recently, frequently pounced on me to remove a live creature, which she deposited in the fire.

As the time got on to about five o'clock I began to worry, and wonder what made my patient so slow. I couldn't account for it in any way; everything appeared quite normal.

Reluctant as I was to do so, I began to feel that I should really have to send for assistance, when I found that in a very short time it would all be over. I never felt so thankful as when that baby was born, but, to my extreme horror, I discovered there was another. Such a thing, I am ashamed to say, had never entered my head, not having ever even seen twins born before.

Nervous and frightened as I was, I told my patient in a complacent manner (as if it was an every-day occurrence to me) that she had got a fine baby boy, and was going to have another.

Poor woman! She wanted one living baby, but not two. Her first thought was that she had no clothing for the second baby; in fact, barely enough for the first. I comforted her as best I could, telling her I could share the clothes till more could be got.

By six o'clock my twins were born—both boys—and everything all right.

I forgot all my discomfort of the day, and went back to the hospital very proud, and very pleased to be able to tell them I had had my first twins.

### Treatment for a Severe Burn.

### EXAMINATION QUESTIONS FOR NURSES.

The question was as follows :—

What would you do in a case of severe burn when the doctor lives several miles away, and may probably not arrive for many hours?

### First Prize.

Severe burns are always accompanied by collapse, therefore this must be treated first. I should put the patient into hot blankets, with plenty of hot bottles in the bed; it is very important that the abdomen and feet should be kept warm. Next give a cup of hot beef-tea or coffee; then if the patient does not show signs of some recovery I should give a little hot brandy and water.

Whilst waiting for my patient to revive somewhat I should prepare strips of lint or old linen soaked in olive oil; next remove the clothing carefully, open any seams if necessary—on no account must the clothes be pulled off roughly, so as to tear or pull on any of the vesicles. I should proceed to dress the wound as I am removing the clothes, so as not to expose too large a surface to the air at a time. Apply a thick covering of wool over the oiled dressing. This should be done with as little fatigue as possible to the patient.

In the case of a small child I should put it at once into a warm boracic bath up to the neck, temperature 98°; it may safely be kept there for some time, providing the temperature is kept up. A bath has the advantage of soaking off the clothing as well as reviving the child. Continue with the hot stimulating drinks until the doctor arrives.

"Belinda."

### Second Prize.

If I were called upon to treat a case of severe burning my first efforts would be directed towards restoring the patient from shock. I should remove the patient to a couch, making him comfortable, with his head low. Warmth should be applied to the surface of the body, and stimulants, such as hot tea, coffee, whisky, administered. Meanwhile I would have oil procured and dressings prepared.

In an emergency any oil will do—sweet, almond, salad, castor, or olive oil. Failing either of these, flour may be dredged thickly over the injuries. The oil may be applied by saturating strips of lint or cotton-wool, or even flannelette.

When the materials are quite ready I would carefully remove the clothes, cutting where necessary to avoid further injury to the wounds.

Should clothing adhere I would cut round it, leaving the piece to be covered by the dressing.

I should be careful not to tear blisters, but should snip them at their lower edges, and gently press out the fluid

I would not expose too much of the injury to the air, as this aggravates shock; but as each small particle of clothing was removed I would apply a strip of lint soaked in oil. When the whole of the injury was covered with the oil dressing I would protect it with antiseptic or ordinary cotton-wool. While I was dressing the wounds I would (supposing I was able to obtain assistance) employ someone attending to the patient. He could administer the stimulants, note pulse and breathing, and watch for a relapse. I would instruct my assistant to refrain from the further use of stimulants on seeing indications of revival.

"Le Petit Enfant."

### Generally Good Answers.

As will be seen from the large number of honourable mentions awarded, the answers on the whole are good, showing a very great improvement upon the standard attained last time this question was put. "Belinda" and "Le Petit Enfant" send answers of nearly equal merit, but "Belinda" takes the first prize because she mentions the advisability of immersing a child in a warm bath. "Le Petit Enfant" shows considerable thought and foresight, and there is great wisdom in advocating the use of flour if no oil is to be had at once. A great many papers found instant sepulture in the waste-paper basket, because they seemed to show an entire absence of knowledge of the fact that shock is the first thing to be dreaded in cases of severe burns.

### Honourable Mention.

This is gained by "Swallow," "Central," "R. J. R.," "Alec," "Jeannie Deans," and "Lanoitan," who all send excellent answers.

### Question for March.

(1) Define the different degrees of danger to life from various kinds of severe burns and scalds. (2) In a long and obstinately slow healing injury from burns state what remedies you would apply and what measures you should take if allowed by the doctor to exercise your own judgment. N.B.—Observe carefully the difference between this question and that of last month.

The Examiner.

### Rules.

The competition is open to all. Answers must not exceed 500 words, and must be written on one side of the paper only, without divisions, head lines, or marginal notes. The pseudonym, as well as the proper name and address, must be written on the *same* paper, and *not on a separate sheet.* Papers may be sent in for 15 days only from the day of the publication of the question. All illustrations strictly prohibited. Failure to comply with these rules will disqualify the candidate for competition. Prizes will be awarded for the best two answers. Papers to be sent to "The Editor," with "Examination" written on the left-hand corner of the envelope.

In addition to two prizes honourable mention cards will be awarded to those who have sent in exceptionally good papers.

N.B.—The decision of the Examiner is final, and no correspondence on the subject can be entertained.

Any competitor having gained three prizes within the current year shall be disqualified from taking another until 12 months shall have expired since the first prize was gained.

### To Nurses.

We invite contributions from any of our readers, and shall be glad to pay for "Notes on News from the Nursing World," "Incidents in a Nurse's Life," or for articles describing nursing experiences at home or abroad dealing with any nursing question from an original point of view, according to length. The minimum payment is 5s. Contributions on topical subjects are specially welcome. Notices of appointments, letters, entertainments, presentations, and deaths are not paid for, but we are always glad to receive them. All rejected manuscripts are returned in due course, and all payments for manuscripts used are made as early as possible after the beginning of each quarter.

### Association for Promoting the Training and Supply of Midwives.

The second annual meeting of the Association for Promoting the Training and Supply of Midwives was held on Friday afternoon at the Caxton Hall. The chair was taken by Mr. A. L. Leon, L.C.C., J.P. (Hon. Treasurer), in the absence of Mr. H. Cosmo Bonsor, who wrote regretting his inability to be present.

After the re-election of the President, Vice-Presidents, and the Council (with the addition of two new members), and the re-election of the Executive and Finance Committees, Mrs. Wallace Bruce, Chairman of the Executive Committee, moved the adoption of the report.

Mrs. Bruce referred to the increased conviction felt by the committee of the necessity for the work that is being carried on and the great extension which could take place if the means at their disposal were adequate to the need. It was to be seen from statistics placed before the meeting, supplied by Miss Wilson, President of the Midwives Institute and member of the Central Midwives Board, that there was a very great shortage of midwives. From figures supplied up to October 1904 it was shown that 10,260 midwives were practising in the past, while only 2,612 were certified or "intending" to practise under the new Act. Despite, however, the shortage, the Association sometimes found it difficult to place those whom they had trained, because frequently a living wage could not be assured the midwife in many districts.

Mrs. Bruce also alluded to the lectures to midwives which had been, and were being, delivered under the auspices of the Education Committee of the London County Council. The Association undertook to visit midwives in London and organise centres. Lecturers were appointed, and apparatus, etc., supplied by the County Council. Eighteen centres were formed, where courses of thirteen lectures, at a cost of 1s. to each midwife, were given, mainly by medical women, to audiences averaging 18 to 20 at each centre. Ten new courses had been started this year; six in the same districts as in 1905. The Training Home at East Ham was doing excellent work; 630 cases had been attended during the year, 15 pupils were trained (three at a time), under the superintendence of Miss Rabson, resident midwife, and the assistant midwife. If funds permitted, many more pupils might be trained. Four hundred and thirty-three women applied for training during the year; 383 were found unsuitable or were not heard of again after the first application, 7 were definitely refused after further inquiry, 26 were accepted, and 17 were under inquiry at the close of the year. The committee had under consideration a plan for making the midwives trained by the Association members of the Association after a certain period of good work, as a means of keeping in touch with them and encouraging a good standard of endeavour in their work. The Association was greatly in need of funds, and a meeting had been arranged for May 17, at Lady Brassey's house, for the purpose of increasing knowledge of the Association, and in this way, it was hoped, obtaining additional subscriptions. London had been apportioned into districts, and ladies had undertaken certain of these districts for the collection therein of £50 a year in annual subscriptions. More helpers were needed in this work.

The report was seconded by Miss Lucy Robinson, who especially referred to the work in East Ham. Miss R. Paget also emphasised the necessity for the co-operation of voluntary bodies in the work of supplying midwives. Miss Broadwood asked if the attendance given at East Ham was free, and on being informed that it was technically free, though many mothers contributed, suggested that a small charge should be made, and that if this were done the institution could be made self-supporting.

The report was adopted, and Dr. Reid, Medical Officer of Health for the County of Staffordshire, was then called upon to address the meeting. He congratulated the Association on the excellent work it had done, and confessed that though he, as many others, realised the need of the Act before it was passed, he had had no conception, until he began to work under the Act, of the appalling conditions prevailing. He supposed that Staffordshire was fairly representative, being chiefly of the working classes and to a large extent rural. Of the 597 midwives who were on the roll, only 26 were trained nurses, and 39 per cent. could not read or write. This year, he was glad to say, 47 of these midwives had retired, and, he hoped, would be replaced by more efficient persons. The Council had in Staffordshire two first-class inspectors, one of whom was a qualified medical practitioner, and Dr. Reid quoted a letter from Dr. Gregg showing the very low class of women with whom she and her co-inspector had to deal. The Council were proposing to organise lectures for midwives, free of cost, and to pay the travelling expenses of midwives attending. In some towns in Staffordshire, Dr. Reid said, the infant mortality was as high as 220 to 250 per 1,000, pointing to the enormous need for improvement. It was hoped shortly to form a Central Association for training midwives in Staffordshire, with an annual grant from the Education Committee.

Miss Wilson, in seconding a vote of thanks to Dr. Reid—which was proposed by Mrs. Bruce—referred to the difficulty of placing midwives in sparsely-populated districts, and spoke in high terms of the system in vogue abroad of State-assured salaries to midwives in such cases.

### Nurses' Social Union.

Three meetings of the Nurses' Social Union have been held in Somerset during the past month. At Minehead the meeting was by kind invitation of Mrs. Bligh—held at her house—and a lecture on "Foods and Feeding" was given by Miss J. M. Joseph, who holds a first-class certificate from the Gloucester School of Cookery. The meeting of the Bristol branch was held by the kindness of the matron—Miss Morris—in the Bristol General Hospital, and about fifty nurses and friends were present. This meeting proved a most enjoyable one. The nurses were shown all over the hospital, and after tea an admirable lecture on Finsen Light, *x*-rays, etc., was given by Dr. Kenneth Wills, followed by a practical demonstration. The lecture was so lucid that even those unfamiliar to the subject could not fail to understand it, and the intent faces of the nurses showed how thoroughly Dr. Wills' wonderful tale enthralled them. A most cordial message of welcome from the committee was much appreciated and it was felt that the meeting had realised one of the aims of the Union that light and leading should be extended from the hospitals to those nurses whose opportunities for knowledge are less. The third gathering was for the Taunton branch at Miss Edow's house in Kingston. Here, too, was novelty, for the lecture on "Nursing in the Tropics" was illustrated by lantern slides. The lecturer, Miss Vicary (late sister of the Radcliffe Infirmary, Oxford), had nursed in most places in the world, and as she added to experience a bright and amusing manner she kept her large audience thoroughly interested, one nurse saying at the end that "she would like to have gone without her tea and listened to Miss Vicary for another hour." That these meetings fill a want is evident; one nurse ran five miles to the town where the brake to Kingston started and had to walk back. She narrowly escaped missing the brake, and on being asked what she would have done in that case, replied "I should have walked on!" Two more centres for Bath and Yeovil have just been formed, so that there will now be no nurse in Somerset beyond the reach of the professional stimulus and friendly intercourse. Miss Amy Hughes has consented to be the first President of the Union.

### Appointments.

[No charge is made for announcements under this head, and we are always glad to receive and publish appointments. The information, to insure accuracy, should be sent from the nurses themselves, and we cannot undertake to correct official announcements which may happen to be inaccurate. It is essential that in all cases the school of training should be given.]

Alexandra Hospital for Children with Hip Disease, Queen Square, London.—Miss Charlotte Cumber has been appointed assistant matron, and Miss Lilian Ryan Jones night sister. Miss Cumber was trained at the Sussex County Hospital; she has since been staff and charge nurse at the David Sassoon Hospital, Poona, India; sister in charge of the women's medical and surgical wards and the operating theatre at the Royal Portsmouth Hospital; and ward sister at the Alexandra Hospital for Children with Hip Disease. Miss Jones was trained at the Royal Portsmouth Hospital, and has since been staff nurse at the Cancer Hospital, Fulham Road, London.

Bethnal Green Infirmary.—Miss Gertrude F. B. Harden and Miss Elizabeth Simpson have been appointed sisters. Miss Harden was trained at Guy's Hospital, where she has since been staff nurse. She has also been on the staff of Guy's Hospital Institution for Trained Nurses. Miss Simpson was trained at Birmingham Infirmary, where she has since been sister. She has also been night sister at the Women's Hospital, Birmingham.

Bruntsfield Hospital, Edinburgh.—Miss A. Wallace has been appointed staff nurse. She was trained at the Children's Hospital, Gateshead-on-Tyne, and the Ingham Infirmary, South Shields.

Central London Sick Asylum, Hendon.—Miss Maud Aland has been appointed sister. She was trained at Islington Infirmary, Highgate Hill, where she was also staff nurse.

General Hospital, Gloucester.—Miss Florence Rayner has been appointed sister. She was trained at Islington Infirmary, Highgate Hill.

Gravelly Hill Workhouse Infirmary.—Miss Emma Jane Watts has been appointed charge nurse. She was trained at Aston Union Infirmary, and has since been assistant nurse at Bristol Union Infirmary and district nurse in Manchester.

Home and Infirmary for Sick Children, Sydenham.—Miss Knowles has been appointed sister. She was trained at Northampton General Hospital, and has since been staff nurse at the Samaritan Free Hospital, London.

Islington Infirmary, Highgate Hill.—Miss Mabel Vidlir has been appointed sister. She was trained at Islington Infirmary, and has since been staff nurse.

Llandrindod Wells Hospital.—Miss A. Bramwell has been appointed matron. She was trained at Charing Cross Hospital and the City of London Lying-in Hospital, and has since been charge nurse under the Metropolitan Asylums Board, night sister at Salop Infirmary, Shrewsbury, and matron of Tredegar Cottage Hospital.

Middlesex County Council.—Miss A. A. Pollard has been appointed inspector of midwives. She was trained at the Brownlow Hill Workhouse Infirmary, Liverpool, where for the past seven years she has held the post of midwife in charge. She has also done private nursing in Edinburgh.

Royal Bath Hospital, Harrogate.—Miss Florence Salisbury has been appointed staff nurse. She was trained at Islington Infirmary, Highgate Hill.

St. Nicholas Home, Byfleet, Surrey.—Miss Florence Williams has been appointed assistant matron. She was trained at the Holborn Infirmary, London, where she was afterwards charge nurse and night superintendent. She has since been head nurse at East Cliff House, Margate, a seaside home for sick children under the Metropolitan Asylums Board.

Southern Hospital, Manchester.—Miss Annie Stuart has been appointed staff nurse. She was trained at the Southern Hospital, Manchester, where she has since been assistant nurse.

Wellesborough Infectious Hospital.—Miss Marion Gertrude Emerton has been appointed nurse matron. She was trained at Leicester Infirmary, and has since been charge nurse at the North Western Fever Hospital, Hampstead, sister at Watford Isolation Hospital, and sister at the Throat and Ear Hospital, Golden Square, London. She has also done private nursing

### Everybody's Opinion.

[Correspondence on all subjects is invited, but we cannot in any way be responsible for the opinions expressed by our correspondents. No communication can be entertained if the name and address of the correspondent are not given as a guarantee of good faith, but not necessarily for publication. All correspondents should write on one side of the paper only.]

### NIGHT NURSES AT WHITECHAPEL INFIRMARY.

"A Whitechapel Nurse" writes: With further reference to the correspondence in your paper respecting the grievances of the Constance Road Workhouse staff, I would like to draw attention to the sleeping accommodation of the night nurses at Whitechapel Infirmary. They do not have one night off duty during the three calendar months, and the rest time is much broken by constant noises. The off-duty time is one half-day per week only, from 1 to 10 p.m., which is really the sleeping time (except from 7 to 10 p.m.), all the relief time allowed. The meals for the staff are provided in the mess-room in a very lax manner, the dinners being often served with unclean cutlery. Moreover, the dietary is often short of the quantities allowed. I may add that though during meal times one is off duty, conversation is restricted. In fact, the whole system of the management and control of the staff certainly needs revising, and attention is most respectfully drawn to these matters which cause unnecessary hardships and discomfort to the staff.

### NIGHT NURSES' HOURS.

"Scot" writes: I have read with some disgust the letters which have recently appeared in your columns in reference to the long hours on duty of night nurses; it seems to me that nurses do very little but grumble nowadays. "One about to go on Night Duty" might have waited until she had had some experience and then aired her views. I am glad she is not one of my staff. I received my training at one of our large provincial hospitals, where there was never a scarcity of work day or night. Since then I have been sister in several hospitals, also night superintendent. I consider myself a fairly competent judge of what a nurse can or should do. I know in the old days we considered the patients, and not ourselves. Grumbling and fretting takes far more out of one than actual work. If probationers would only give the matter a thought, they would see that those in authority, having been through the mill themselves, fully realise what they are asking their nurses to do. I have never yet come across a Matron who looked upon her nurses as mere machines, to get as much out of them as possible. I know of nurses who have been taken off duty for the most trifling ailments, and treated with every kindness. It is utter selfishness on a nurse's part to wish to come off duty unless there is good cause for it. We all know what one short means to our fellow-workers. If a night nurse has any method at all, except under exceptional circumstances, she can find some time for herself without neglecting those under her care. Few wards are always heavy; sometimes it is one ward and then in another, giving each nurse a little time to recoup her strength. For my part I do not see that the life on night duty is one of complete self-denial, and even if it is, why brag about it? It is only part of the life we have voluntarily agreed to lead. I do not consider 12 hours on duty too long, considering the period during which the term of night duty usually lasts.

"One Who Wishes to Help" writes: I was glad to see that some kind friends have come to the front on behalf of nurses and their long hours. In most hospitals and nursing homes there are boards of governors who are supposed and believed by the general public to see that the nurses are fairly dealt with; but, alas! these gentlemen are more often than not a one-sided party who stand by the Matron or Lady Superintendent whether she be right or wrong, and never stretch out a helping hand to the unfortunate nurses. I have several friends in the nursing profession who relate most awful tales of their experiences both in hospital and at private nursing, but of these two I have come to the conclusion that the latter is much the worse. In hospital, although the hours are long, there is a regulated time for going on and off duty; but in private nursing there is no rule whatever as to having proper rest and going out to fresh cases in rotation. For instance, one of my friends who had a very trying time with a patient for several weeks returned to the nursing home, and finding several nurses there, made up her mind to have a good night's rest. She asked and obtained leave to retire, but was afterwards surprised to receive orders to be ready for a long journey the same night. She very naturally made some remark about being sent out so soon again, but the only excuse given by the Matron was that she thought my friend "the most suitable nurse for the case." Now this meant being out of bed and having no rest whatever for about forty hours, for she had been up the previous night with her patient, had travelled for five hours that day, and the train journey the same evening (besides a long car drive) took over four hours. She had to remain up with her new patient that night, and was on duty till the doctor came at 12.30 p.m. the next day. The result of this long strain and overwork was a bad attack of neuralgia, which lasted for months. Although she complained several times of not feeling well, there was no notice taken or any proposal made to take her off duty. Now, why do not the governors step in and make a rule that nurses are not sent out without a proper amount of rest, say at least 24 hours, except when specially asked for, or on account of religion?

"Shamrock IV." writes: "A. L." is getting rather mixed, and decidedly away from the point. What I wished to say is that 12 hours of unbroken duty at night are too much for a nurse. "A. M. M. B." tries to defend those who are the cause of giving us 12 hours by stating that there is not much to do—in fact, simply to watch sleeping patients and listen for calls—and evidently thinks that the three hours when the day nurses are "buzzing around" is quite a cheerful time for a nurse fatigued with the nine hours' duty. My own experience of 15 years' nursing is that there is plenty to do, and even when it comes to "only watching and listening," who does not know the strain of the lonely watching, and the moments when one feels that they could give all they possess to lose themselves in sleep, if only for a moment? If I can, then I bathe my face and behind my ears with cold water, or try to get a cup of tea or coffee, put my head out of the window, and do various other things to keep myself awake. "A. L." says she has done six years' duty, and it has not demoralised her. Of course duty does not demoralise; but when one is worn out with hard work, long hours, need of proper sleep, insufficiency of fresh air, loss of appetite, or not enjoying one's food, which is as bad, it is impossible to do one's duty properly. No doubt my nursing days are nearly over, but for the sake of thousands of my "sisters" in the work I sincerely hope that they will all get more rational hours of duty, and, for those who need it, quiet sleeping quarters, good, well-cooked food on well-laid fresh dainty looking small tables with nice surroundings. It only requires a good manager for the home and a little more money for a few extra nurses.

"M. E. H." writes : In your interesting paper the subject of night duty has been much discussed for the past two or three weeks. There seems to be a diversity of opinion. The majority are against 12 hours night duty as being too long. May I be allowed to point out one or two things which seem to be a secondary consideration, but which, in my opinion, should be first. One point to be taken into account is whether the nurse takes her food well, also the amount of sleep she gets. Some nurses—they are in the minority I admit—sleep as well in the day as the night. I myself, for example. It is the rule in many hospitals that if the nurse cannot take the ordinary fare no effort is made to find out what she can take. For instance, if a nurse cannot eat meat she might be able to eat fish or some other substitute. In the case of night nurses these are details which should be looked into. A nurse who sleeps well and takes sufficient food can do 12 hours' night duty for three months, or even a longer period, without any injury to her health. I know of more than one large training school where the term of night duty is 12 months. I need scarcely say that at the end of the term the nurses require a holiday, and many of them, to use their own words, "are never the same." Again, in some hospitals there is a relieving nurse, who helps in the busy wards, and the night nurse thereby escapes the "morning rush," so well known to most of us; also a night off every month is allowed. An opinion was expressed in your columns that there was nothing to do for the first three hours. I wonder where that fortunate nurse did night duty. Four hourly, and even two hourly, treatment is a familiar term to all of us, and the various feeds, foments, temperatures, and medicines cannot all be done while the clock strikes ten. In my first three hours, in an accident ward, I have on more occasions than I could count admitted four patients, all to be washed, and usually dirty, besides attending to minor accidents and the wants of the patients in the ward. I am of the same opinion as "M. R." that "there is very little time for sewing or reading, and sometimes not time to get a cup of tea." Then again, I have often noticed that there seems a sort of feud between day and night nurses. Something is found out of place in the morning. The day nurse exclaims, "Oh! that is the night nurse," in a tone that implies nobody; but the night nurse would not do such a thing. I emphatically say that night nurses are entitled to every consideration. The long hours on duty may be difficult to alter without inconvenience to the hospital, but we can at least make the work as light as possible. Undisturbed rest during the day is more the exception than the rule, and a nurse not feeling rested cannot put her heart into her work, although she is expected to do so under any circumstance. My night duty is a thing of the past, but I retain vivid recollections of trying to divide my attention between several patients all dangerously ill. As one of your correspondents remarked, a nurse has, unfortunately, "nerves" like other people, and the anxiety and responsibility tend to lessen the mental as well as the physical faculties.

### OVERWORKED PROBATIONERS.

"Feminine" writes : I could picture "X. Y. Z." so vividly as I read her letter in your issue of to-day's date—strong in mind and body, so strong that she has missed a woman's womanly sympathy, enjoying that rude health which exacts as its price the lack of "the fellow-feeling which makes us wondrous kind." Not knowing what illness is for herself, she will not, unless she be absolutely compelled to do so, recognise it in any but those who are patients already acknowledged as being ill. A woman whose subordinates would "die rather than complain of sickness" to her, who, if by any chance she had to notice some "trifling ailment," instead of believing an ounce of prevention to be worth a pound of cure, would denounce the poor unfortunate owner of the "cold, headache or bad finger" as "whining or neurotic." And then, supposing it should happen to be serious illness, her "most loving care and attention" will consist of an assurance that it is entirely through carelessness that the illness has been brought about, generally making the invalid wish she had never been born to commit such a crime as being laid up. I cannot, like "X. Y. Z.," look back on twenty years ago, but my experience extends over half that time, during which I expected and had "hard work, rough accommodation, and the plainest of fare," and have never minded it, but the difference lies in that I have heard many grumbles, not at any one of those things, but at the lack of sympathy and the injustice of the treatment received at the hands of some who, unfortunately for their own womanliness, and also for the happiness of those who work under them, have apparently entirely forgotten that in days gone by they too were "pros."

### THE DISTRICT NURSE AND RHEUMATISM.

"L. D." writes : Referring to the "Nurses' Clinic" on the "District Nurse and Rheumatism," may I suggest that the pain accompanying the slightest movement of any joint in "acute rheumatism" would be considerably lessened if "many-tailed flannel bandages" were used instead of rollers? The latter cannot be adequately applied unless the limb is considerably elevated, and this amounts to intense pain for the patient. Rollers may keep a little more securely, but during the early stages the sufferer is only too glad to avoid unnecessary movement. Also, I have found liniment of belladonna very soothing, and it is frequently ordered by doctors to be sprinkled on the wool to be wrapped round the joints. This can be prepared and laid beneath the joint with the bandage, thus making one movement sufficient, and that only slight elevation. Also, I think that the cleansing with turpentine rather extreme treatment until the pain lessens. Surely one should remember, and try to save needless suffering when no serious issues are at stake, and it is perfectly certain that the patients would appreciate thoughtfulness for their comfort in this respect. I quite agree with the writer that daily sponging is very soothing, but let it be done with as little movement, and as quickly as possible, avoiding all swollen and tender parts.

### NURSES AND THEIR GRIEVANCES.

"Old Westminster" writes : I often wonder what Florence Nightingale, my dear old Matron, Miss Pyne, and such women would think if they read the pretty constant grumbling letters of the nursing profession. I feel sometimes positively ashamed when outsiders look into my "Mirror" and know how predominate "self" is among us, who of all women are looked upon as leading unselfish lives. There seems to be an earnest desire not to do as much as we can, but as little; and to our shame it is said that a number of nurses will not be long together before either their food, or their housing, or their pay, or their bad time generally are under discussion. I look back on my 15 years of nursing with great thankfulness. Certainly they have been the happiest part of my life, and I am looking forward to many more like years. My health has been good, though my holidays have been few. I have done a lot of night duty, but though it is hard and tiring work I do not think that 12 hours on duty is too much to be balanced by 12 hours off duty, for a reasonable length of time—*i.e.*, three months. A woman with ordinary health should be able to do this without much ill effect, and I think that those women who have to be so very careful about their own health are better out of the nursing profession. The exercise of a little common sense on the subject of hot baths at any and all times, thin shoes and stockings, and tea and biscuits just before meal times would considerably lessen the tendency to "headaches and colds," and as to bad fingers—in the days of my training bad fingers were considered almost on a par with bedsores! The best nurses are the most loth to report themselves sick. I do not mean that "nervous headache going on for months," or anything else that "goes on" wrongly, either our own health or anything that unnecessarily annoys us; but I think complaints made in the right way to the proper person seldom get ignored. That "cup of tea" in the nurses' rooms before bed I blame for much sleeplessness, and often, too, the tea or coffee served before bed might with advantage be replaced by a glass of ale or stout. It would not only induce sleep and appetite, but prevent much of that habitual constipation from which many night nurses suffer, and give a better tone generally to the whole system. Night duty is hard, because it is unnatural, but it is not harder than many other things women do, nursing or not nursing. Moreover, there is a beautiful and a good work to be done in the night; the pain and the misery and the sin-sickness is realised more in the darkness than in the light, and it is left to us, if we will it so, to make it our business not only to put on that poultice or that icebag, but to say ever such a little word of comfort or advice, or do some small thing not on the duty list, which may just influence for good years of a hitherto wasted life. Our influence, whether we wish it or not, must carry either for good or evil, especially in the night season.

### BEAUTIFYING THE PATIENTS.

"F. A. H." writes: A little while ago the front-door bell of a hospital in the North of England was rung, and when it was answered, outside was found a thinly-clad little girl of about eight years old and her brother of six wheeling a perambulator with a boy of three years old in it. Upon the receiving nurse inquiring what they wanted, the girl said, "Please, he's broke his leg, and mother's sent this letter to say will doctor please look to him." The child was taken in, and in due course the quilt he was wrapped in and his little nightdress were returned to the perambulator, and off went the two who had brought it, looking very satisfied with themselves for the piece of business they had accomplished. About two days after they came to inquire about Willie, and nurse asked how their mother was. "Mother ain't in bed," said the wee maiden; "it's my big brother what's got bronchitis and plumbonia, and mother has to poultice him about every ten minutes, and that's why she can't come." No children visitors are allowed in our wards, but when Willie was able to be up in the wheel-chair his sister came and begged hard to see him. She looked so dreary in her very old clothes that nurse's heart was touched, and she wheeled Willie out into the corridor so that he might be seen. The little woman caught hold of nurse's arm and said, "Oh, my! don't he look bonnie"? When she had seen him she ran off in great glee to tell her mother, who this time was herself ill in bed, all about it. It was principally soap and water which had done so much towards the beautifying of Willie, but probably his clean rosy cheeks and neatly brushed hair were ornaments unknown to the small sister.

### THE ADMISSION OF FRIENDS AS PROBATIONERS.

"E. B." writes: With reference to the question of "E. M. H.," "Should Friends be Admitted as Probationers?" I have had several years' experience in the nursing profession, and cannot see why any objection should be made, providing that they are suitable in every other way—refined, intellectual, and sympathetic. Surely such friends should be a help to each other as they enter upon a career, with all its difficulties and failures—which are many, especially to the girl who perhaps leaves home for the first time. "Sister" and "nurse" intend to be kind and help; still it is all so strange, and the "new pro." feels often very lonely, but if she had a friend in the hospital with whom she would have so much in common, how it would cheer her and brighten many a weary hour! I believe that many a girl who would have been a credit to the profession, and has given up nursing, would never have done so if she had possessed at the commencement of her nursing life a true friend, who in her days of disappointments would have given her words of encouragement.

### Novelties for Nurses.

(By Our Shopping Correspondent.)

### NURSES' CLOAKS.

(Matthew Rose and Sons, Mare Street, Hackney, London, N.)

Now that the days are getting longer and brighter, many nurses will probably be discarding the cloaks that have seen such hard service and are now unable to face the sunlight. In that case they could not do better than go to Messrs. Matthew Rose and Sons, Mare Street, Hackney, who make a speciality of nurses' cloaks. Here are to be seen several new models expressly fitted for the spring season. There is the "Reading," a smart cloak with circular cape, detachable velvet collar, and—a great boon—a roomy pocket beneath the cape. The material is very fine and light, guaranteed rainproof and incapable of fading. The price is only 21s.—a real bargain! Then there is the same shape in heavier material at 25s. 9d. There is also a "Reading" in a very pretty steel grey, the material of which is excellent, and the price 29s. 9d. A brown "Reading" in heavier material is 35s. 9d. All these cloaks are thoroughly well finished both inside and out, and have a very good cut about them. Rather a novelty in cloaks is one made with a honeycomb yoke, which hangs very well, and has a stylish look about it. It is made in light, spring material, and its price is 23s. 9d. There is also a plain black circular cloak with yoke at 21s., and a black cloak with three capes at 21s.; while the plain circular cloak so beloved by some nurses costs only 18s. 9d., and a coat can be had for 15s. 11d.

Cloaks are not the only things that nurses will find at Messrs. Rose's; in fact, all necessary parts of a nurse's equipment are to be had. Cotton materials for dresses are both moderate in price and good in quality. A dark blue with narrow white lines at 7¾d. a yard is excellent value. Then there are shoes, made in soft glacé, so restful to tired feet, with special ventilation under the sole and rubber heels. A plain shoe with a strap can be had for 6s. 11d., and with a little more ornamentation at 9s. 11d. There are bonnets to suit all purses and all ages. A very neat little bonnet, having just the plain brim turned back with velvet, is made in all colours at 5s. 11d. The "Phyllis" with a bow of velvet costs 8s. 9d., and a full bonnet for more elderly nurses, with a folded front, is 12s. 11d. with veil. There are various collars and cuffs, and a gored apron with bib and pocket at 1s. 11½d.

### TRAVEL NOTES AND QUERIES.

By our Travel Correspondent.

Acommodation in Cornwall (A Private Nurse).—Many thanks for your valuable information. It is all filed for reference. You will hear from me.

Somewhere for Mountains and Flowers (Crediton).—You will not find flowers so early as you expect. In April and May they begin in their beauty, and I think you could not do better than St. Jean-de-Luz, close to the Spanish frontier. I can give you addresses if you decide to go there.

Holiday at St. Malo (Matron).—Many thanks for your very kind letters. Many seek our help, but very few remember to write and thank us for information received, and to express satisfaction with the results.

Rules in Regard to Correspondence for this Section.—All questioners must use a pseudonym for publication, but the communication must also bear the writer's own name and address as well, which will be regarded as confidential. All such communications to be addressed "Travel Correspondent, 28 Southampton Street, Strand." No charge will be made for inserting and answering questions in the inquiry column, and all will be answered in rotation as space permits. If an answer by letter is required, a stamped and addressed envelope must be enclosed, together with 2s. 6d., which fee will be devoted to the objects of "The Hospital" Convalescent Fund. Ten days must be allowed before an answer can be published.

### A Book and its Story.

### THE CHAMPAGNE BRAND.*

In the brightly written chapters which form the book issued under the title of "The Champagne Brand," Mrs. John Lane, who is an American, makes observations on the manners and customs of the two nations to which she is allied, from the point of comparison. Common sense, dry humour, and a smart style combine to make this collection of fugitive ideas so attractive that one can open it at random and be sure of coming upon something novel and arresting. At the outset, be it known, the champagne standard is a non-alcoholic one, and used merely as another term for vulgar ostentation. The author expresses her opinion as follows: "There is nothing in the modern world so absolutely real and convincing and universal as its pretence. It has set itself a standard of aims and living which can best be described as the champagne standard. To live up to the champagne standard you have to put your best foot foremost, and that foot is generally a woman's. . . . It is the women who have set the champagne standard. A man who lays a great stress on the importance of trivialities has either a worldly woman behind him, or he has a decided feminine streak in his character." Women, worldly and otherwise, will agree to the wisdom of further remarks on the same subject: "Yes, it is the champagne standard; for nothing so accurately describes the insincere pretensions and frothy striving after one's little private unattainables. It is aspiration turned sour." But, on the other hand, aspirations, when directed to higher and less selfish ends, are the very life of noble deeds. For, as the author continues: "Aspirations real and true keep the world progressive, make of men great men, and of women great women." The lesser aspirations which occupy lesser minds and those circumstances which embitter the existence of greater ones are summed up as follows: "It is the minor aspirations after what we have not got, what the accident of circumstances prevents us from having, which make of life a weariness and a profound disappointment. Not the tragedies of life make us bitter, but the pinpricks."

In quest of ancestors the members of the great Republic cross the Atlantic to give an added lustre to a democratic house, whose fortune has been made in the humbler walks of commerce.

"There is a growing mania in America in these days for ancestors. It is a luxury that can be indulged in only after people have accumulated money. . . . The Heralds' College could tell many a queer story of our sturdy Republicans in search of forbears. An Englishwoman told me that a New York family had annexed a Crusading forefather of her own, as well as one who had his head chopped off, and to whom they had no more right than the grocer round the corner. . . ." "You are funny Republicans," she added genially, "coming over here and grabbing our ancestors." The author affirms that she finds no one so frank as a frank Englishwoman. The one in question continues her comments: "What is the use of celebrated ancestors," she added, "if your whole present family is dull as ditchwater and bore you to distraction? I'd swop my Crusading ancestor and my chopped-off-head one any time for a cousin with brains. But, mind you, I don't want your American millionaires grabbing 'em without leave."

Quite one of the best stories in the book is told of a certain American lady called Susan. It is too long to quote fully, but summarised it reads thus: "There are the Bedfords of New York. Susan and I went to school together. Hitherto she had put on no airs with me, for I know the family traditions, and that her excellent father began life as a cobbler. Then he forsook cobbling and started a corset manufactory, which was a distinguished success, because he invented a bone so like the whale's that even a clever fish could not have told it was not his, and the deception made the old man's fortune. In the corset period Susan married a 'drummer' in the business, and it was left to Susan to do the aspiring, for she realised that it was not possible for her husband to accommodate himself to the growing grandeur. She passed a sponge over their previous existence, and every time I saw them in New York she had added a new lustre to their glory. The last time the door was opened by an English butler. I saw at once that the whole family were in terror of him." When accompanied to the front door in American fashion by her friend, conscious of the strong disapproval of the English butler, she caught sight of a satin banner. "Our coat of arms," explained Susan by way of introduction. "Just come home. It cost a great deal; everything costs so much. We have the same arms as the Duke of Bedford. It is pleasant to have a Duke in the family." Her friend intervenes. "Come in here Susan," and I led her into her own parlour, for I did not wish to lower her in the estimation of that noble being who was preparing his mighty mind to show me out. "Listen to me; you and Joe haven't any more to do with the Duke of Bedford than the cat's foot. Besides, his name is not Bedford, but Russell. For goodness sake don't make such an idiot of yourself." "I guess," said Susan, who was deeply offended, "I guess that young man at Tiffanys knows more about it than you do. He engraves for the first families, and he says it is all right."

In "Gunpowder or Tooth-powder" Mrs. Lane expresses her conviction that it is a national failing that so little attention is paid by the average Britisher to the condition of his teeth and those of his children. "The English have not the habit of going to the dentist; money paid to him they consider wasted—there is nothing to show for it. . . . They still have their teeth taken out rather than stopped (filled), as being cheaper, and when they are all out they replace them on too slight a provocation by what American humour calls 'store teeth.' . . . If England wants good war material (and there has been some adverse criticism of the quality of her soldiers) she must cultivate it, and it is her duty to step in where the parent fails." As a means to this end the author suggests that the Board Schools should provide toothbrushes for the pupils, and there should be a daily morning exercise which should take precedence of lessons, namely, a sort of toothbrush drill, in which everyone should begin with a vigorous toothbrushing as a preliminary precaution and a necessary preservative of sound health. On the other hand, "one of the most promising signs of the future of the American people, among others that are the reverse, is the importance they attach to good teeth. . . ." If we believe Mrs. Lane a terrible doom is in store for the English nation: "So in due course England will lose her proud position as the greatest nation in the world, simply because England would not go to the dentist, which is a curious neglect for a people whose morning tub is much less likely to be neglected than their morning prayers."

---

* By Mrs. John Lane. (John Lane. 6s.)

### Notes and Queries.

**REGULATIONS.**

**The Editor is always willing to answer in this column, without any fee, all reasonable questions, as soon as possible.**

**But the following rules must be carefully observed.**

1. **Every communication must be accompanied by the name and address of the writer.**
2. **The question must always bear upon nursing, directly or indirectly.**

**If an answer is required by letter a fee of half-a-crown must be enclosed with the note containing the inquiry.**

*Maternity Work in the South of France.*

(177) Could I get private work as a maternity nurse in the South of France or in Italy under an English doctor, and to whom should I apply? I should be willing to sign for two or three years. I hold the Q.C.H. and C.M.B. certificates.—*E. M. H.*

Unless you have introductions you would not obtain work, and we do not think there is any satisfactory opening such as you desire. Certainly in Italy you would be useless without a knowledge of the language spoken. Why not apply to one of the nursing homes, such as the Nice Nursing Institute, Villa Pilatte, Avenue Désambrois, Nice, or St. Paul's Home, Via Palestro, Rome?

*Nursing in Paris.*

(178) Can you give me the addresses of nursing homes in Paris where they take English nurses? I am fully trained and speak French a little. Also how could I obtain an appointment in Canada or any of the Colonies?—*Anxious.*

The Holland Nursing Institution, 25 Rue d'Amsterdam, and St. George's Nurses' Association, 92 Rue de la Boche, both employ English nurses. You might hear of work in Canada by writing to the Lady Superintendent, the Victorian Order of Nurses for Canada, 578 Somerset Street, Ottawa, Ontario, Canada. For the West African nursing service write to the Hon. Secretary of the Colonial Nursing Association, Imperial Institute, London, S.W. Almost all the Colonies are sufficiently supplied with nurses, and your chances without good introductions are small.

*Handbooks on Physiology.*

(179) I wish to become a nurse, but at present have duties at home. What books on physiology would you recommend me to study, and which is the best medical dictionary?—*Cymri.*

"Elementary Physiology for Nurses," price 2s.; and "The Nurse's Dictionary of Medical Terms and Nursing Treatment," price 2s. These can be had from the Scientific Press, 28 and 29 Southampton Street, Strand. The last-named book will give you all you need; ordinary medical dictionaries are very expensive.

*Young Woman of Feeble Intellect.*

(180) Can you tell me of any home where a young woman of feeble intellect could be placed, either free or for a very small payment?—*M. W.*

Write to the Secretary of the National Training Home for the Feeble-minded, 36 King William Street, London, E.C.

*Home for Winter Months.*

(181) Can you tell me of some comfortable home where I could spend the winter months? The charges must be very moderate, as I cannot afford to pay more than about £1 per week.—*A. C.*

We do not reply by letter unless 2s. 6d. is sent. See rules. Also reply to *M. M. G.*

*Home for Paralysed Lady.*

(182) Can you give me names of any homes where a paralysed lady could be received on payment of a guinea per week? She is very helpless, and suffers from incontinence of urine. She has to be dressed every day, but can walk with a little assistance, is able to use her hands, and is a very happy, contented old lady, requiring little attention after she is up.—*M. M. G.*

There are doubtless many nurses who would be glad to receive such patients, and the proper course is to advertise.

**Handbooks for Nurses.**

| | Post Free. |
|---|---|
| "How to Become a Nurse: How and Where to Train." | 2s. 4d. |
| "Nursing: its Theory and Practice." (Lewis.) ... | 3s. 6d. |
| "Nurses' Pronouncing Dictionary of Medical Terms." | 2s. 6d. |
| "Complete Handbook of Midwifery." (Watson.) ... | 6s. 4d. |
| "Preparation for Operation in Private Houses." ... | 0s. 6d. |

Of all booksellers or of the Scientific Press, Limited, 28 & 29 Southampton Street, Strand, London, W.C.

### For Reading to the Sick.

THE SILKEN CORD.

Poor indeed thou must be, if around thee
Thou no ray of light and joy canst throw;
If no silken cord of love hath bound thee
To some little world, through weal or woe;
If no dear eyes thy tender love can brighten,
No fond voices answer to thine own;
If no brother's sorrow thou canst lighten
By daily sympathy and gentle tone,
Daily struggling, though enclosed and lonely,
Every day a rich reward will give:
Thou wilt find by hearty striving only,
And truly loving, thou canst truly live!

*Anon.*

"Silver and gold have I none," said St. Peter to the lame man at the Beautiful Gate, "but such as I have give I thee." And what he gave was infinitely better than gold or silver would have been. He said to him, "In the name of Jesus Christ of Nazareth, rise up and walk." Then, taking the lame man by the hand, he lifted him up; and at once his weak limbs became strong, so that he could walk alone, needing no longer to sit by the temple entrance and ask for alms. Better help had been given him than any alms the poor man ever received.

This story is a parable as well as a fact. Its lesson is, that there are better things to give than gold and silver. If we can put new life and hope into the heart of a discouraged man, so that he rises out of his weak despair, and takes his place again in the ranks of active life, we have done a far better thing for him than if we had put our hands into our pockets, and given him money to help him nurse a little longer his miserable and unmanly despair. The truest sympathy is not that weak emotion which only sits down and weeps with a sufferer, imparting no courage or hope, but that wiser love, which, while it is touched by his pain and grief, and feels tenderly toward him, seeks to put new strength into his heart, to enable him to endure his suffering in a victorious way.

Sympathy is better than money: so is courage, so is cheer, so is hope. It is better always to give ourselves than to give our money: certainly we should give ourselves with whatever else we may give. "The gift without the giver is bare." Christ Himself gave no money; but every life that came near to Him in faith went away enriched and helped. He gave love, and love is the brightest and richest coin minted in this world. And all of us can give love: none are too poor for that.—*T. R. Miller.*

A clasp of hands will oft reveal
A sympathy that makes us feel
Ourselves again; we lose our care:
And in our heart's first glad rebound
At tender sympathy new found,
The world once more seems bright and fair.

## Figures and Tables

**Fig. 9. f1:**
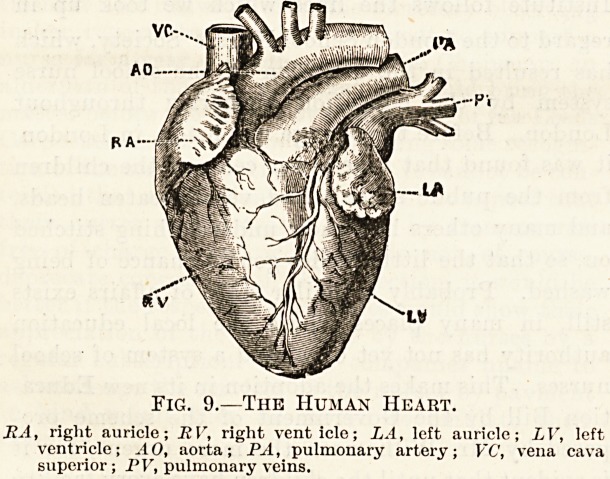


**Fig. 10. f2:**